# Carbon-Nanotube Microelectrodes for Electrochemical Determination of Melatonin

**DOI:** 10.1002/elan.202400191

**Published:** 2024-08-21

**Authors:** Neeraj Kumar, Nilni E. Weerawarna, Noe T. Alvarez

**Affiliations:** Department of Chemistry, University of Cincinnati, Cincinnati USA

**Keywords:** cross-section microelectrode, cyclic voltammetry, highly densified carbon nanotubes, melatonin, square wave voltammetry

## Abstract

Voltammetric methods hold promise for the rapid and sensitive quantification of melatonin. This study reports the direct electrochemical quantification of melatonin using carbon nanotube (CNT) fiber cross-sections as microelectrodes. Six identical highly densified CNT fiber cross-sections were employed to quantify melatonin in the range of 0.05–100 μM. The limit of detection and quantification were 10 and 35 nM, respectively, with a sensitivity of 0.1322 nA/μM. Interference studies with uric acid, hypoxanthine, and ascorbic acid demonstrate its performance. Real-world application was highlighted by measuring melatonin in food, pharmaceutical, and human urine samples.

## 1 | Introduction

Melatonin (*N*-acetyl-5-methoxytryptamine, Mel) is a hormone primarily released from the pineal gland, with smaller amounts also produced in the retina and gastrointestinal tract [1, 2]. Mel regulates the circadian rhythm of the body by maintaining the biological sleep-wake cycle [3]. Mel, an indoleamine, is produced by L-tryptophan metabolism through serotonin and N-acetylserotonin. In this pathway, the rate-limiting steps are catalyzed by the enzymes hydroxyindole-*o*-methyltransferase and arylalkylamine *N*-acetyltransferase [4]. The Mel level in healthy humans varies depending on age, ranging from a few pM to ~1400 pM. In addition to ageing, factors such as exposure to light decrease Mel secretion in the human body [5–9]. Mel is associated with sleep efficiency [10, 11], mood regulation [12], retinal physiology [7, 13], immunologic functions [14], seasonal affective disorder [15, 16], sexual maturation, and reproduction [17, 18] as well as being an effective antioxidant, anti-inflammatory, and neuroprotective agent [19, 20]. As a dietary supplement or medicine to regulate circadian rhythm disorders, Mel is available in controlled-release (oral dosage: 10 mg) and immediate-release (oral dosage: 0.2–10 mg tablets and 1–20 mg capsules) formulations [21–23]. Mel is not under the regulatory control of the FDA. However, according to the American Academic of Sleep Medicine (AASM), Mel appears to be safe for treating delayed sleep–wake phase disorder, jet lag, insomnia, and shift work disorder [21, 23].

Although Mel has long been considered an animal hormone, specifically a neurohormone, it has also been identified in invertebrates, algae, bacteria, fungi, and plants. In plants, Mel acts as an antioxidant or growth promoter that protects tissues from oxidative stress and adverse environmental conditions [24]. Mel is found in more than 140 different plant species, fruits, wine, and foods [25, 26] at concentrations varying from picograms to micrograms per gram or milliliter. Consequently, endogenous Mel concentrations may be influenced by food intake. Food items rich in Mel include cherries, olive oil, grape wine, walnuts, almonds, beans, tomatoes, and grains [26].

Several studies have suggested that the short-term use of Mel is safe, even at high doses, with only mild adverse effects such as headache, dizziness, and sleepiness. However, owing to its easy availability, Mel uptake has increased, with some individuals self-medicating at high doses. This behavior increases the risks associated with high Mel levels, including irregularities in biological and physiological functions [27]. Although studies have suggested that long-term Mel use has mild effects, adverse effects can occur, especially in children, adolescents, and pregnant and breastfeeding women. In particular, Mel transferred into breast milk can significantly affect nursing infants, causing daytime sleepiness and sedation [28, 29]. Therefore, Mel detection is important for both the early diagnosis of Mel-related side effects and controlling the adverse effects of high Mel doses on various health conditions.

Numerous analytical techniques, including capillary electrophoresis [30, 31], capillary electrophoresis with electrochemical detection [32], high-performance liquid chromatography [33, 34], gas or liquid chromatography-mass spectroscopy [35, 36], and immunoassays [37, 38] have been utilized for the estimation of Mel. Despite offering selective and sensitive analysis, these techniques have low spatial resolution and require sophisticated and expensive instrumentation, time-consuming sample preparation, and skilled personnel. In contrast, electrochemical techniques, which have great potential for miniaturization, offer excellent sensitivity across a wide concentration range, are fast, reproducible, easy to operate, and portable, making them suitable for on-site analysis [3, 39, 40]. In recent years, Voltametric methods such as cyclic voltammetry (CV) [41], square wave voltammetry (SWV) [17], differential pulse voltammetry (DPV), and amperometry [42] using both unmodified and modified electrodes have been applied for Mel determination. In particular, SWV and CV have been widely applied for the electrochemical detection of analytes. CV can be used to study the electron-transfer kinetics and redox behavior at the electrode surface, whereas SWV allows the quantitative determination of electroactive analytes. Recently reported electrodes for Mel detection include glassy carbon electrode (GCE) modified with a nanopalladium polymer nanocomposite [17], a protein nanodot-gold nanoparticle (AuNP) polymer nanocomposite [43], AuNPs-multiwalled carbon nanotubes-3-*n*-propyl-(4-methylpyridinium) silsesquioxane chloride [44], and 2D-MoS_2_ with cucurbit[8]uril [45]. Simpler system, such as carbon fiber electrode with fast scan cyclic voltammetry (FSCV)[46], the boron-doped diamond electrode (BDDE) with CV[47], BDDE with DPV and amperometry[48], and the carbon fiber electrode with DPV [49] have also been used for the detection of melatonin. However, some modified electrodes, consisting of nanocomposite materials deposited on substrates such as GCEs, indium tin oxide, and carbon paste electrodes, typically require tedious and time-consuming preparation processes or provide poor sensitivity and selectivity in complex matrices.

Herein, we report a microelectrode based on the cross-sections of highly densified carbon nanotube (HD-CNT) fibers for the direct electrochemical sensing of Mel. Owing to their unique properties, including excellent electrical properties, fast electron-transfer kinetics, a wide potential window, and a high surface-to-volume ratio, CNTs have been used to fabricate sensors for various applications [50–56]. The alignment of CNTs into a fiber allows the exploitation of the extraordinary properties of CNT cross-sections. CNT fibers have demonstrated higher electrical conductivity, higher signal-to-noise ratios, and lower detection limits than CNT arrays, CNT towers, and dispersed or arranged CNTs [57–59]. As the cross-sections of HD-CNT fibers have high electrochemical accessibility, excellent electrical conductivity, and high stability, they are suitable as working electrodes for the direct estimation of electroactive analytes. Our research group has studied the properties of HD-CNT fiber for the electrochemical sensors, which has also been utilized for the detection of heavy metal ions [60, 61]. In this study, a CNT forest was grown on a Si wafer by chemical vapor deposition (CVD) using a Fe—Al catalyst. Subsequently, spinnable CNT fibers were extracted, densified, and used to fabricate cross-section microelectrodes. The developed HD-CNT-fiber-based cross-section microelectrodes were suitable for the quantification of Mel without any modification despite of interferants present in solution.

## 2 | Experimental

### 2.1 | Chemicals and Materials

Sodium hydroxide (NaOH), sodium phosphate monobasic dihydrate (Na_2_HPO_4_·2H_2_O), sodium phosphate dibasic dihydrate (NaH_2_PO_4_·2H_2_O), ascorbic acid (AA), uric acid (UA), Mel, and hypoxanthine (HX) were obtained from Sigma Aldrich (St. Louis, MO, USA). Phosphate buffer (μ~1), which was used as a supporting electrolyte, was prepared using NaOH, H_3_PO_4_, Na_2_HPO_4_·2H_2_O, and NaH_2_PO_4_·2H_2_O. Spinnable CNT arrays were synthesized via CVD using ethylene as a carbon source (Wright Brothers, Cincinnati, OH, USA), a Fe—Al catalyst (Goodfellow Corporation, Pittsburgh, PA, USA), and Ar as a carrier gas. CNT-embedded polymer films were fabricated using an Embed-812 embedding kit (Electron Microscopy Science, Hatfield, PA, USA). All other solvents and chemicals were analytical grade, and Milli-Q water (18 MΩcm) was used to prepare stock solutions.

### 2.2 | Instrumentation

Electrochemical studies were performed using a PalmSens4 electrochemical workstation (Houten, Netherlands) with a three-electrode setup. The HD-CNT-fiber cross-section microelectrode with six cross-sections was used as the working electrode, a Pt wire as the counter electrode, and Ag/AgCl (3 M KCl) as the reference electrode. The surface morphologies of the HD-CNT fiber and cross-section electrode were characterized using field emission scanning electron microscopy (FE-SEM, FEI XL30, Hillsboro, OR, USA) operated at an acceleration voltage of 10 kV. To investigate the quality of the CNT fibers, Raman spectra were recorded using an inVia Raman microscope (Renishaw, Wotton-under-Edge, UK) with a 633 nm Ar-ion laser. For the vertically aligned CNT array, Raman spectra were collected using an acquisition time of ~10 s with 5% and 10% power (Figure S1).

### 2.3 | Carbon Fiber Preparation

**FIGURE 1 |:**
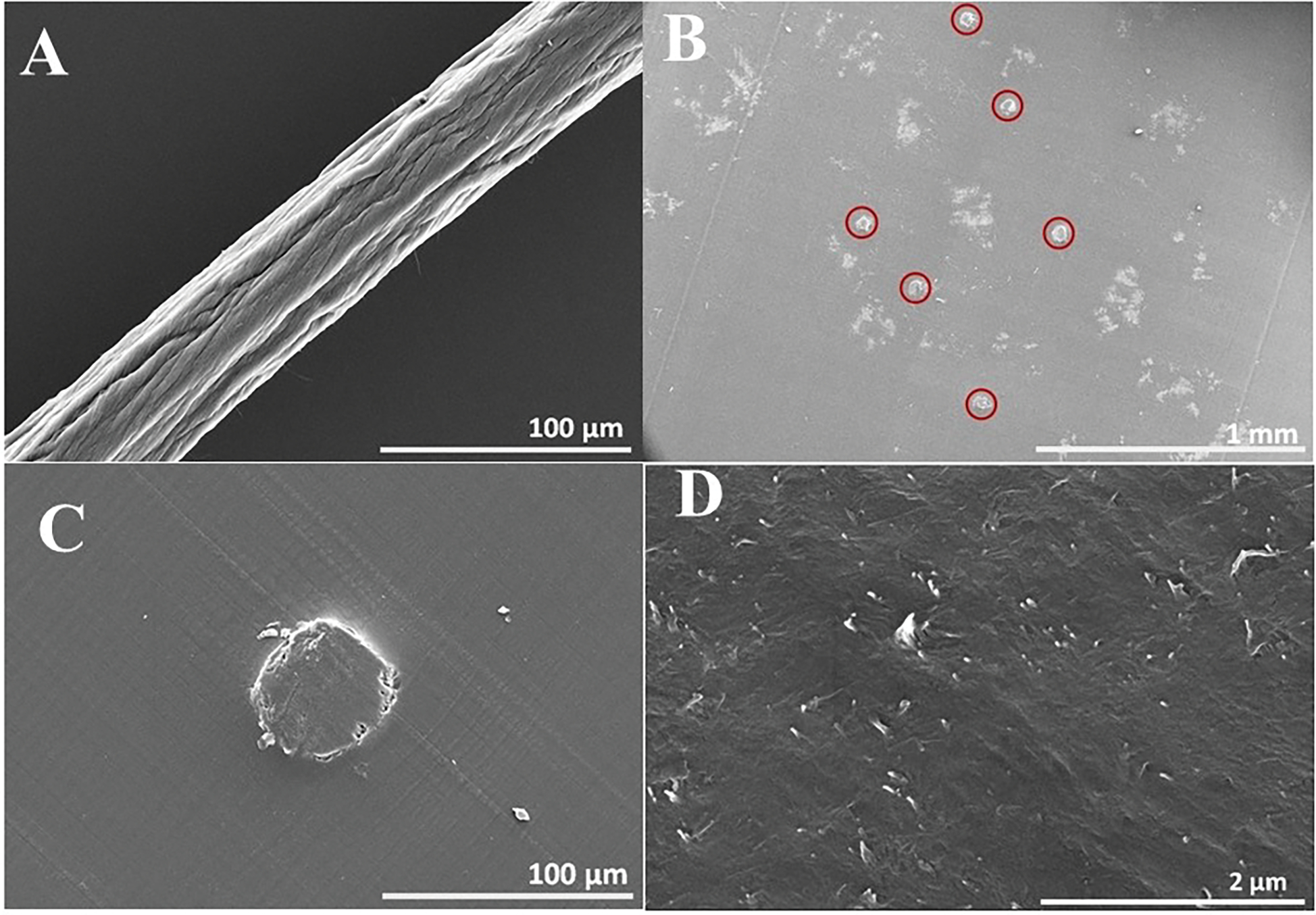
SEM images of open-ended CNT fiber microelectrodes. (A) a densified CNT fiber (~48 μm diameter) at magnification ×500, (B) six identical HD-CNT-fiber cross-sections embedded in a polymer at magnification ×50, (C) a HD-CNT-fiber cross-section microelectrode at magnification ×500, and (D) an HD-CNT-fiber cross-section microelectrode at magnifications ×2500.

CNT fibers with a diameter of ~48 μm were obtained from vertically aligned CNT forest arrays produced by CVD, as previously reported [62, 63]. The CNT fibers were prepared by pulling and twisting the vertically aligned CNT array from one end while simultaneously applying a spinning and pulling motor. Owing to van der Waals forces, the fibers exhibited poor packing density. To overcome this issue, the fibers were densified by soaking in acetone under optimized conditions (96 h at 30°C). The densification process results in good conductivity and enhances the alignment of the CNTs within the CNT fiber [62, 63]. An SEM image of the acetone-soaked HD-CNT fiber (~48 μm diameter) is shown in Figure 1A.

We recently reported the detailed process for fabricating cross-section microelectrodes using HD-CNT fibers [60, 61]. Briefly, six identical HD-CNT fibers (~48 μm diameter, 1.5 cm length) were arranged parallel to each other on a tape support. The HD-CNT fibers were placed in a 2 mL capsule-shaped plastic vial, which was then filled with a monomer mixture (EMbed-812) and cured at 90°C in an oven for 24 h. After extraction from the plastic vial, perpendicular microtome cuts were made through the polymer-embedded CNT fiber, producing slices with a thickness of 40 μm. This process produced 40 μm long HD-CNT fibers with open ends on both sides of the sliced film. Figure 1B shows the six identical HD-CNT-fiber cross-sections, demonstrating the high-density packing of CNTs within the fiber. The exposed open ends of the six HD-CNT fibers (~48 μm diameter) on one side of the sliced film were connected to a conductive metal wire via silver paste and sealed with epoxy resin for electrical insulation. The other side of the film, which also contained six HD-CNT open-end cross-sections, was applied as a cross-section microelectrode for the electrochemical estimation of Mel. Figure 1C and D show SEM images of the HD-CNT-fiber cross-section microelectrode at different magnifications. The optical image of HD-CNT-fiber cross-sections microelectrode employed for melatonin detection is shown in picture of Figure S2.

### 2.4 | Electrochemical Detection of Mel

A Mel stock solution (1.0 mM) was prepared in Milli-Q water. Phosphate buffer (pH 7.25, 0.42 M) was used as a supporting electrolyte for electrochemical investigations. For Mel analysis, the required amount of stock solution was added to an electrochemical cell containing 2 mL of phosphate buffer (pH 7.25), and the total volume was made up to 4 mL using Milli-Q water. For CV studies the following conditions were used: t-equilibration, 2 s; initial potential, 0.1 V; potential vertex 1, 1.0 V; potential vertex 2, 0.1 V; scan rate (*v*), 100 mV/s; and potential step, 25 mV. For SWV studies, the following parameters were used: equilibration time, 20 s; initial potential, 0.2 V; final potential, 1.2 V; frequency (*f*), 10 Hz; amplitude, 25 mV; and potential step, 4 mV. After each test, the microelectrode was regenerated by applying a potential of −600 mV for 120 s in phosphate buffer (pH 7.25). The electrode was stabilized by recording five consecutive cycles in phosphate buffer (pH 7.25).

### 2.5 | Food Samples

Mung beans were purchased from a local market. Ten bean seeds were soaked in Milli-Q water overnight, crushed, and then mixed with 2 mL of phosphate buffer and 2 mL of Milli-Q water. The obtained solution was filtered and then used for the analysis of Mel. For this analysis, the mung bean sample was spiked with various known concentrations of Mel (2.49–74.78 μM).

### 2.6 | Pharmaceutical Samples

To prepare pharmaceutical samples, two Mel-containing Natrol (3 mg/tablet) or Walgreens (10 mg/tablet) tablets were weighed and then ground into a fine powder using a mortar and pestle. Subsequently, a 0.5 mM stock solution was prepared by dissolving the required amount of powder in Milli-Q water. The stock solution was filter by filter paper (Fisher brand, Fisher Scientific). These stock solutions (0.5 mM) were used to prepare solutions with 5–25 μM concentrations, for which the voltammetric response was recorded.

## 3 | Results and Discussion

### 3.1 | Effect of Scan Rate During CV Analysis

**FIGURE 2 |:**
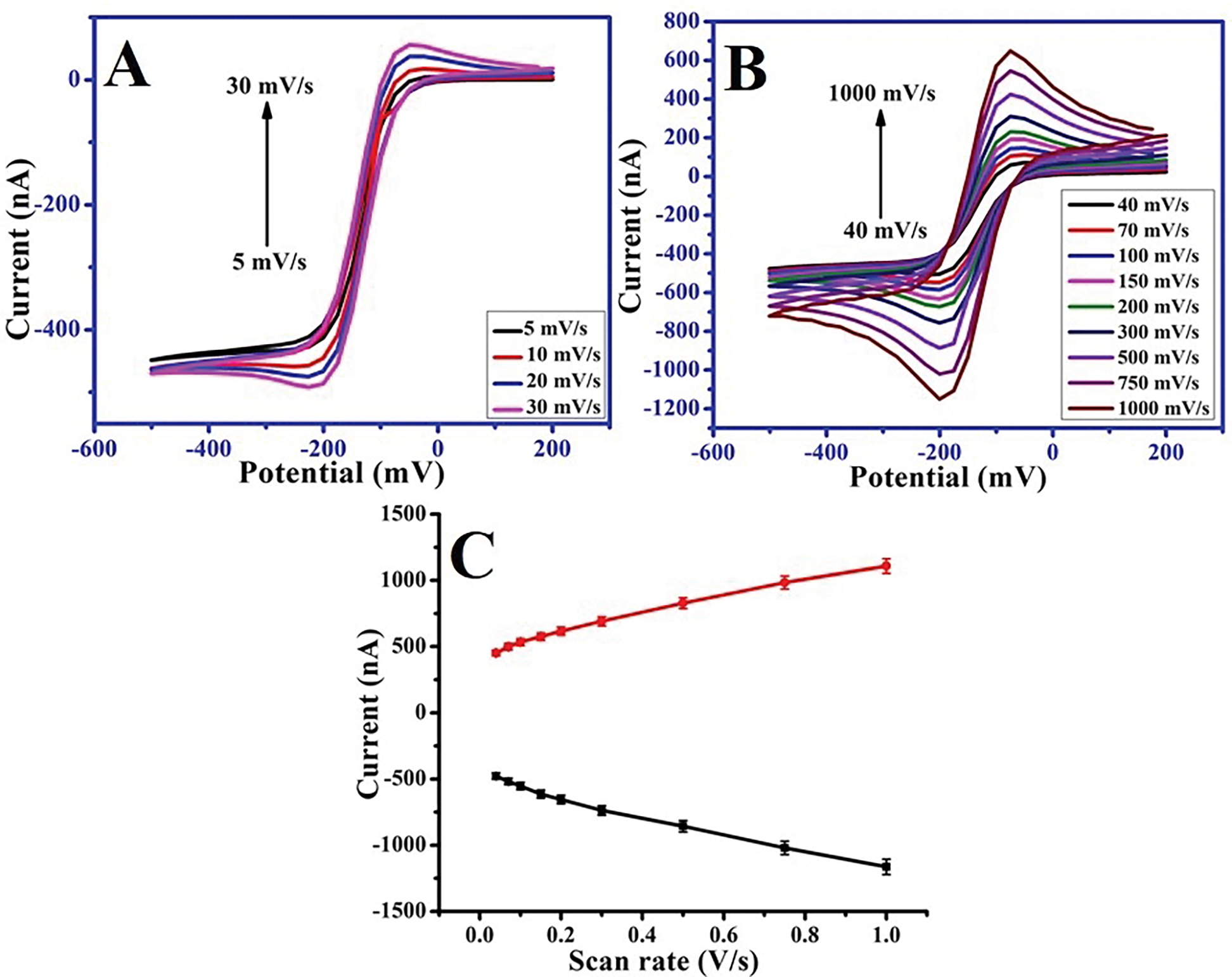
Cyclic voltammograms recorded for 5 mM Ru(NH_3_)_6_ (prepared in 50 mM KCl) solution at scan rates of (A) 5–30 mV/s and (B) 40–1000 mV/s (from lowest to highest peak current). (C) Dependence of cathodic and anodic peak currents on scan rate.

The effect of the scan rate on the cyclic voltammograms recorded using the HD-CNT-fiber cross-section microelectrode was investigated using a redox analyte of 5 mM [Ru(NH_3_)_6_]^3+^ prepared in 50 mM KCl. At a lower scan rate of 5 mV/s, a sigmoidal steady-state cyclic voltammogram was observed for the reduction of [Ru(NH_3_)_6_]^3+/2+^ (Figure 2A), which is characteristic of hemispherical diffusion at a cross-section microelectrode. The limiting current ($i_{lim}$) of a disk-type of microelectrode[64] is given by the following equation:

$$i_{lim}=4nFDaC$$

where n is the number of the electrons involved in the redox reaction, D is the diffusion coefficient (8.2×10^−6^ cm^2^/s), F is the Faraday constant (96485 C/mol), a is the radius of the cross-section or disk-shaped microelectrode (24 μm), and C is the concentration of [Ru(NH_3_)_6_]^3+^ in the reaction solution (5 mM).

The observed liming current (~472 nA) was ~2 times higher than calculated limiting current (~227 nA) for the electroactive species (Figure 2A). The higher current can be due to contribution of side walls of the HD-CNT rods which are uncovered during the microtome process and high value of the limiting cathodic current was observed compared to theoretical surface area. A shift from hemispherical to planner diffusion began at a scan rate of 40 mV/s (Figure 2B), with a peak observed for [Ru(NH_3_)_6_]^3+/2+^. Cyclic voltammograms recorded at scan rates up to 1000 mV/s are also shown in Figure 2B. At higher scan rates, well-defined cyclic voltammograms with reduction and oxidation peaks were obtained. Linear relationships were observed between the reduction or oxidation peak current and the scan rate at 40–1000 mV/s (Figure 2C), as represented by the following equations:

$$i_{pa}=707.067\;v\;+\;454.70,{\;R}^{2}=0.976$$

$$i_{pc}=-735.179\;v\;-\;478.365,{\;R}^{2}=0.979$$

where $R^{2}$ is the regression coefficient, $i_{pa}$ is the anodic peak current (nA), $i_{pc}$ is the cathodic peak current (nA), and v is the scan rate (mV/s). These linear relationships indicate that electron transfer at the HD-CNT-fiber cross-section microelectrode is an adsorption-controlled process.

CV was also used to investigate the nature of the electrochemical reaction at the microelectrode surface with Mel as an analyte. The influence of the scan rate on the peak current for the electro-oxidation of Mel was examined by recording cyclic voltammograms for 10 μM Mel in phosphate buffer (pH 7.25) at scan rates of 25–300 mV/s. A well-defined oxidation peak was observed for the electro-oxidation of Mel at the microelectrode surface. Furthermore, the oxidation peak current of Mel increased with increasing scan rate (Fiures S3 and S4). The plots of peak current ($i_{p}$) vs. scan rate (v) and $log\left(i_{p}\right)$ vs. log(v) were linear, as represented by the following equations:

$$i_{p}=0.023\;v+2.0736,{\;R}^{2}=0.9917$$

$$\log\left(i_{p}\right)=0.5339\;log(v)-0.394,{\;R}^{2}=0.9945$$

The slope of the $log\left(i_{p}\right)$ vs. log(v) plot was greater than 0.5, indicating that the electrochemical oxidation of Mel at the HD-CNT-fiber cross-section microelectrode was adsorption controlled [17].

### 3.2 | SWV Analysis

#### 3.2.1 | Effect of Concentration

**FIGURE 3 |:**
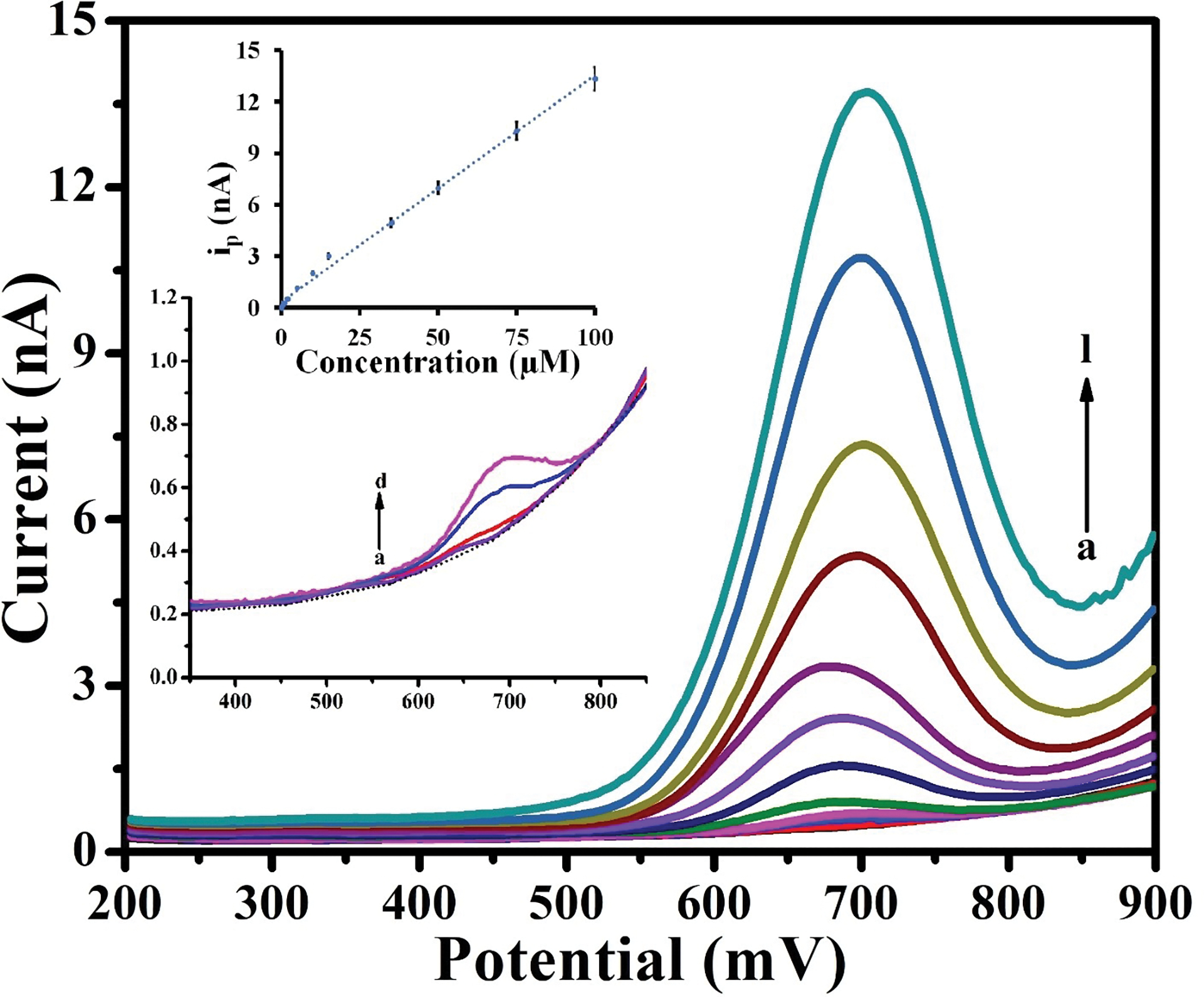
Square wave voltammograms recorded for (a) 0.05, (b) 0.1, (c) 0.5, (d) 1, (e) 2, (f) 5, (g) 10, (h) 15, (i) 35, ( j) 50, (k) 75, and (l) 100 μM melatonin in phosphate buffer (pH 7.25) using the HD-CNT-fiber cross-section microelectrode. The upper inset shows the corresponding calibration plot, and the lower inset shows the square wave voltammograms at lower concentrations. The blank is represented by a dotted line.

The HD-CNT-fiber cross-section microelectrode was used to quantify the concentration of Mel using electrochemical methods. To construct a calibration plot, the change in the oxidation peak current as a function of Mel concentration was examined using SWV. Before SWV data collection, the 20 s equilibration time allows voltammogram data collection with reduced noise. Figure 3 shows the SWVs recorded for Mel at concentrations of 0.05–100 μM in phosphate buffer (pH 7.25). A sharp oxidation peak was observed at ~670 mV vs. Ag/AgCl for Mel oxidation at the HD-CNT-fiber cross-section microelectrode surface. As shown in the inset of Figure 3, the peak current increased linearly with the Mel concentration. The linear regression equation is as follows:

$$i_{p}=0.1322\;[0.05-100\;\mu M]+0.3376,{\;R}^{2}=0.9956$$

These observations indicate that the developed HD-CNT-fiber cross-section microelectrode can be employed to detect low concentrations of Mel. The statistical parameters obtained for Mel determination are summarized in Table 1. The limit of detection (LOD) and limit of quantification (LOQ) were calculated as 3σ/b and 10*σ/b, respectively, where σ is the standard deviation of three consecutive scans (S/N = 3) recorded in blank phosphate buffer (pH 7.25) and b is the slope of the calibration plot. The LOD and LOQ determined for the HD-CNT-fiber cross-section microelectrode (10 nM and 35 nM, respectively) are considerably lower than those of previously reported electrochemical sensors (Table 2).

BDDE: boron-doped diamond electrode; GCE: glassy carbon electrode; f-MWCNT: functionalized multiwalled carbon nanotubes; AuNP: bold nanoparticles; Si4Pic^+^ Cl^−^: 3-*n*-propyl-(4-methylpyridinium) silsesquioxane chloride; PLL: polylysine; PNDs: protein nanodots; AHNSA: 4-amino-3-hydroxy-1-naphthalene sulfonic acid; PdNPs: palladium nanoparticles; ErGO: electrochemically reduced graphene oxide

#### 3.2.2 | Effect of Phosphate Buffer pH

**FIGURE 4 |:**
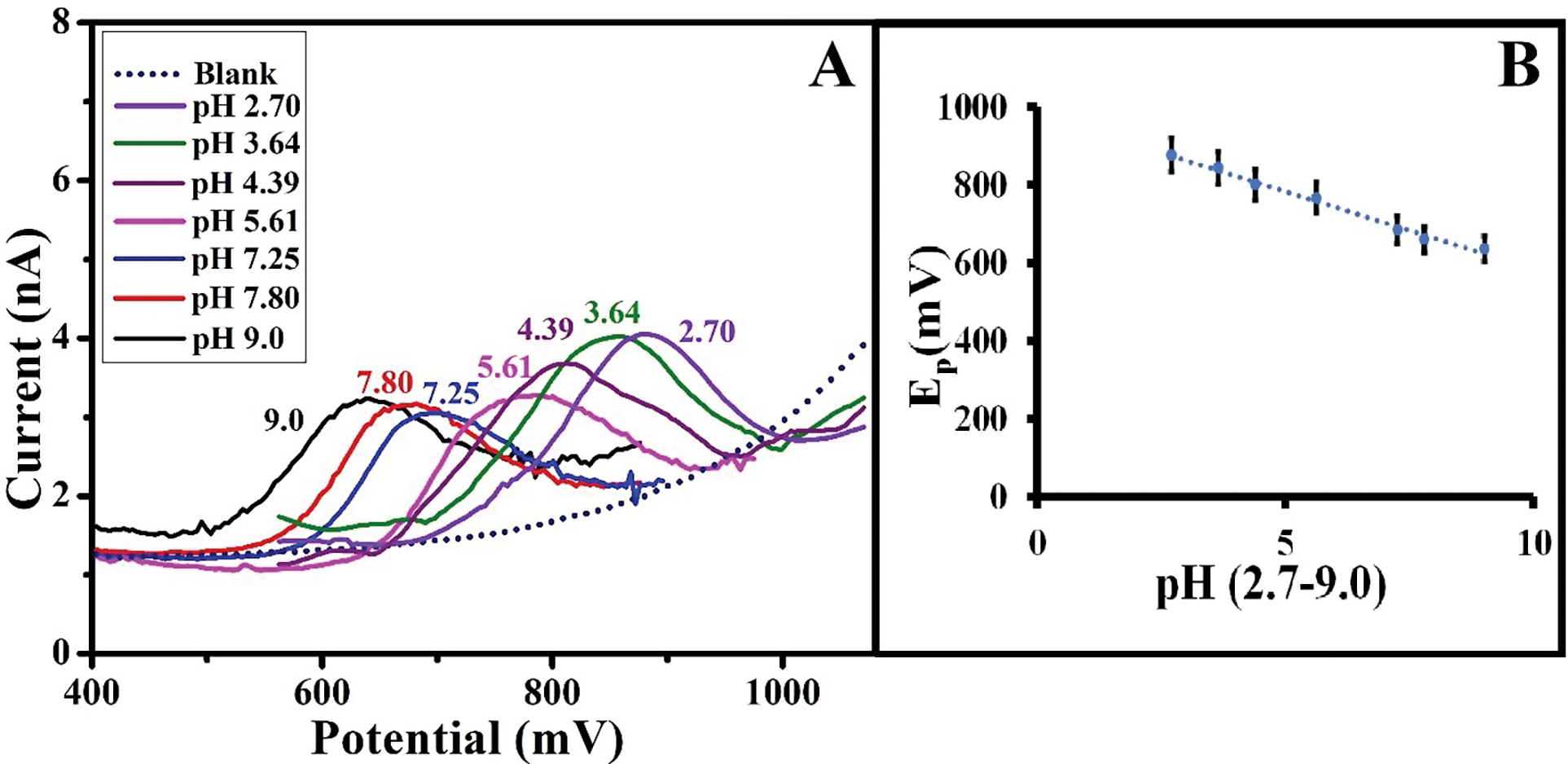
(A) Square wave voltammograms recorded for 10 μM melatonin in phosphate buffer solutions with varying pH values (2.7–9.0). (B) Corresponding calibration plot.

The effect of pH was investigated to elucidate the electrochemical oxidation mechanism of Mel on the surface of the HD-CNT-fiber cross-section microelectrode. Square wave voltammograms were recorded for 10 μM Mel in phosphate buffer solutions with pH values of 2.7–9.0. The electro-oxidation potential of Mel shifted towards a more negative potential upon increasing the pH of the phosphate buffer (Figure 4).

The electro-oxidation of Mel at the HD-CNT-cross-section microelectrode was assumed to exhibit Nernstian behavior, as represented by the following equation:

$$E\left(mV\right)=E-59\;n\;log\left[H^{+}\right]$$

**SCHEME 1 |:**
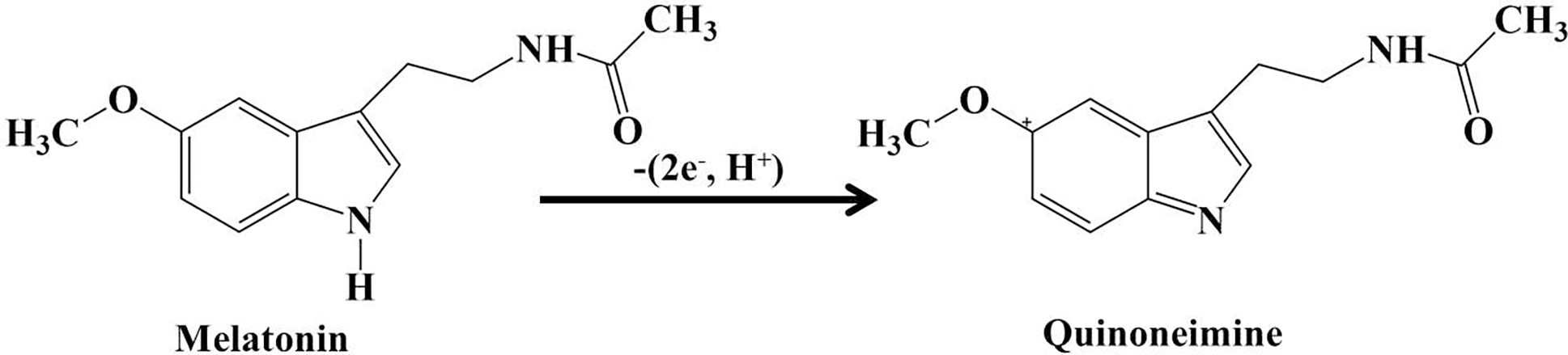
Mechanism for the electrochemical oxidation of melatonin at the HD-CNT-fiber cross-section microelectrode.

According to this equation, the slope of the E vs. pH plot should be 0.059 (for n = 1). The dependence of the electro-oxidation peak potential of Mel on the pH of the supporting electrolyte can be expressed by the following linear equation:

$$E_{p}=-40.019\;pH+984.35,\;{\;R}^{2}=0.9897$$

where $E_{p}$ is the electrochemical oxidation potential of Mel. The slope (${dE}_{p}/dpH=-40.019$) suggests that an unequal number of electrons and protons participate in the electro-oxidation of Mel, consistent with previously reported values [3]. The proposed mechanism for the electro-oxidation of Mel at the microelectrode surface is shown in Scheme 1. The optimized pH value of 7.25 was used for all subsequent electrochemical analyses.

Laviron’s equation was also used to calculate the number of electrons that participated in the electro-oxidation of Mel [65]. The oxidation of Mel at the HD-CNT-cross-section microelectrode was a completely irreversible adsorption process, which can be described by the following formula:

$$E_{p}=E^{0}+(RT/\alpha nF)\left[ln\left({RTk}_{s}/\alpha nF\right)-lnv\right]$$

where $E^{0}$ is the formal potential, $E_{p}$ is the peak potential, R is the gas constant (8.314 J/(mol·K)), F is the Faraday constant (96498 C/mol), T is the thermodynamic temperature (298.15 K), $k_{s}$ is the standard rate constant, n is the number of electrons transferred, and α is the electron-transfer coefficient (assumed to be 0.5 for an irreversible process [66]).

As shown by the cyclic voltammograms recorded for 10 μM Mel at scan rates of 5–300 mV/s (Figure S2), the peak potential increased with increasing scan rate. The change in the peak potential with scan rate can be expressed by the following equation:

$$E_{p}\;=\;0.0222\;lnv\;-\;15.1591$$

The number of electrons involved in the electro-oxidation of Mel was calculated as n = 2.31 (~2). Thus, these results indicate that two electrons participate in the electro-oxidation of Mel at the HD-CNT-fiber cross-section microelectrode, as shown in Scheme 1.

#### 3.2.3 | Interference Study

**FIGURE 5 |:**
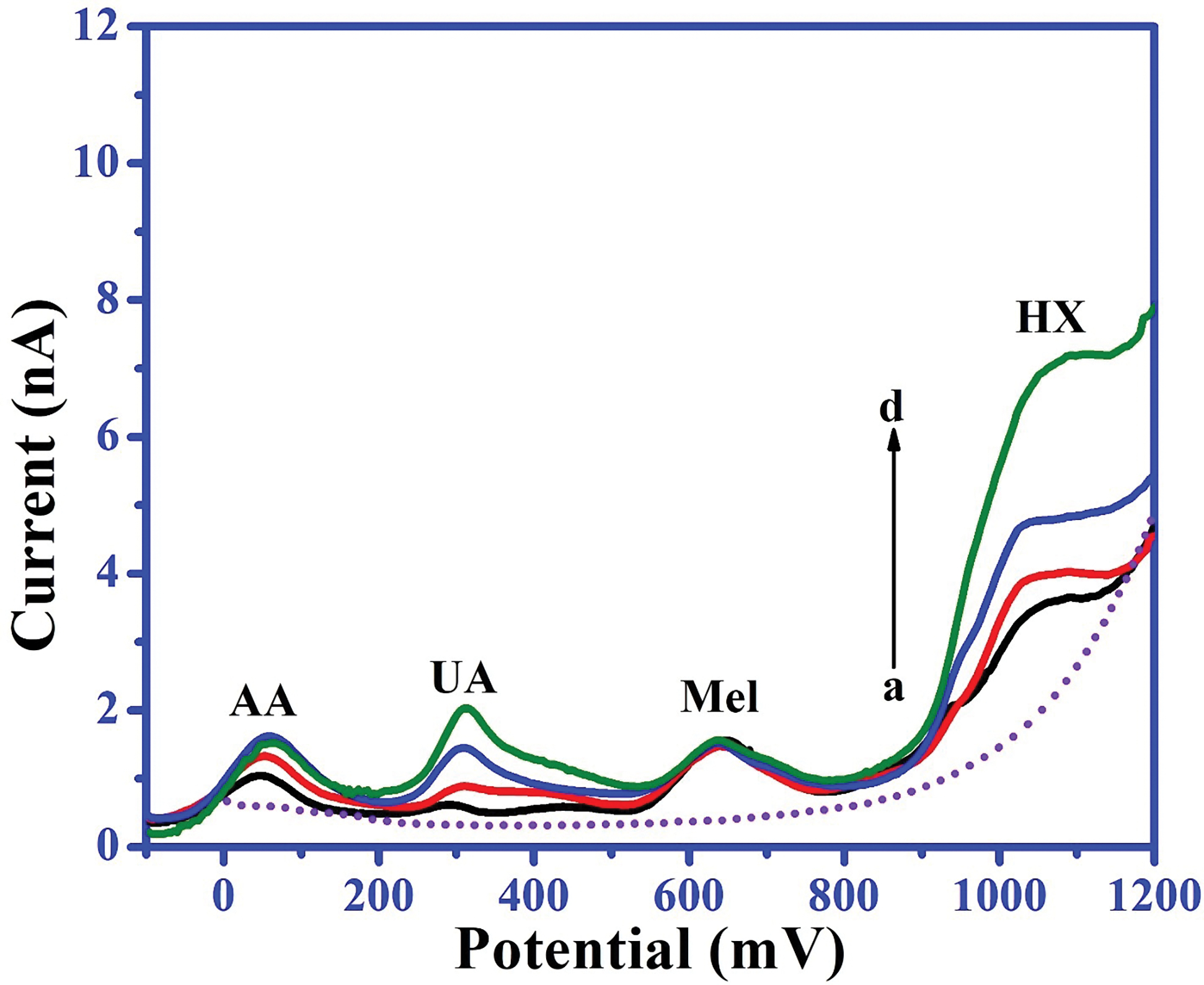
Square wave voltammograms recorded for (a) 10 μM AA + 5 μM UA + 5 μM HX, b) 20 μM AA + 10 μM UA + 10 μM HX, c) 30 μM AA + 15 μM UA + 15 μM HX, and d) 50 μM AA + 25 μM UA + 25 μM HX with 5 μM melatonin in phosphate buffer (pH 7.25).

The selective determination of an analyte in complex matrices is a key challenge for practical applications. To address this challenge, Mel was analyzed in a complex matrix containing interfering molecules such as AA, UA, and HX, which are commonly found in body fluids. As high concentrations of these molecules can potentially interfere with the oxidation of Mel, thereby affecting its estimation, interference studies were conducted under optimized conditions. Square wave voltammograms were recorded for a fixed concentration of 5 μM Mel with varying concentrations (up to 200 μM) of UA, AA, and HX (Figure 5). Notably, the electrochemical oxidation of Mel was unaffected by the presence of UA, AA, and HX, even at concentrations 10 times higher than that of Mel. These results suggest that the HD-CNT-cross-section microelectrode can be applied for the electrochemical detection of Mel in the presence of such interfering substances.

### 3.3 | Real Sample Analysis

#### 3.3.1 | Food Sample Analysis

The practical applicability of the developed HD-CNT-fiber cross-section microelectrode was investigated by the estimation of Mel in food samples. Square wave voltammograms were recorded for mung bean samples spiked with various known concentrations of Mel. The gradual addition of Mel to the test solution resulted in an increase in the peak current. A calibration plot constructed using the Mel concentration and peak current was used to estimate the Mel concentration in the mung bean sample (Figure S5). The Mel concentration was found to be 3.4 μM, which is in good agreement with the previously reported concentration of Mel in mung beans.

#### 3.3.2 | Urine Sample Analysis

To investigate the practical utility of the presented microelectrode for analyzing biological fluids, concentration of Mel was determined in urine samples. Before analysis, the collected urine sample was diluted 5 times using phosphate buffer (pH 7.25) to reduce the complexity of the solution. The diluted sample was spiked with various known concentrations of Mel (1 mM stock solution) and square wave voltammograms were recorded. Two oxidation peaks were observed at approximately 330 and 680 mV, corresponding to UA and Mel, respectively. The peak current of Mel increased as the UA concentration increased. The Mel concentration was back-calculated using the calibration equation for the peak current, and the obtained results are summarized in Table 3. The recovery was found to be greater than 99% with a relative standard deviation (RSD) of ±3.41%. These results demonstrate the practical applicability of the developed HD-CNT-fiber cross-section microelectrode for real sample analysis.

#### 3.3.3 | Pharmaceutical Sample Analysis

The developed HD-CNT-fiber cross-section microelectrode was applied to the detection of Mel in commercially available tablets (Natrol and Walgreens). Square wave voltammograms were recorded for sample solutions with different concentrations of Mel, and the concentrations of Mel in the tablets were calculated using the calibration plot, as summarized in Table 4. The observed values are in good agreement with the standard Mel dosages of the tablets.

### 3.4 | Stability and Reproducibility

The stability and reproducibility of the HD-CNT-fiber cross-section microelectrode were investigated using 10 μM Mel in phosphate buffer (pH 7.25) over 30 days. The electro-oxidation peak current of Mel remained unchanged for 20 days, with an RSD of ±1.21%. After 20 days, the changes in the electro-oxidation current increased slightly, with an RSD of ±3.46%. The intraday reproducibility of the developed sensor was determined by collecting five square wave voltammograms at 1 h intervals for the 10 μM Mel solution. The obtained RSD value of ±1.21% (for n = 5) demonstrates the high accuracy and reproducibility of the employed cross-section microelectrode.

## 4 | Conclusion

HD-CNT fibers, fabricated by densifying the CNT fibers obtained using the CVD method, were used to fabricate an HD-CNT-fiber cross-section microelectrode. The CNT array, HD-CNT fibers, and cross-section electrode were characterized using Raman spectroscopy and FE-SEM. The HD-CNT-fiber cross-section microelectrode was used for the electrochemical detection of Mel. The studied sensor was successfully applied for the qualitative and quantitative analysis of Mel in the range of 0.05–100 μM, with an LOD and LOQ of 10 nM and 35 nM, respectively. Advantageously, the fabricated cross-section electrode was highly stable and required no modification for the electrochemical detection of Mel. Furthermore, the cross-section electrode exhibited no interference with common interfering molecules such as UA, AA, and HX. Even during real sample analysis, the reported sensor could continuously monitor Mel at high UA concentrations of up to 25 μM. The practical applicability of the developed sensor was demonstrated for food (mung beans), pharmaceutical, and human biological samples (urine). Notably, a spiked recovery study in human urine samples gave a recovery within the acceptable range of 99–102%. Therefore, it is concluded that HD-CNT-fiber cross-section microelectrode are suitable for the detection of Mel in real samples.

## Supplementary Material

Suplementary Information

Supporting Information

Additional supporting information can be found online in the Supporting Information section at the end of this article.

## Figures and Tables

**TABLE 1 |:** Statistical parameters for estimation of melatonin.

Validation parameter	Result

Concentration	0.05–100 μM
Sensitivity (μA/μM)	0.1322±0.0027
Correlation coefficient (R^2^)	0.9956
Intercept	0.3376±0.113
LOD	10 nM
LOQ	35 nM

**TABLE 2 |:** Comparison with some previously reported methods for the electrochemical determination of melatonin.

Electrode/technique	Concentration range	Limit of detection	Year	Reference

BDDE, SWV	0.5–4 μM	0.11 μM	2012	[67]
AHNSA:PdNPs:ErGO/GCE, SWV	5–100 μM	0.09 μM	2016	[17]
GCE, SWV	5–200 μM	0.32 μM	2017	[68]
Graphite-automotive varnish, SWV	10–100 μM	0.49 μM	2021	[69]
f-MWCNT-AuNP-Si4Pic^+^Cl^-^/GCE	4.9–55.5 μM	1.6 μM	2022	[44]
AuNP-PLL/PNDs/GCE, SWV	0.1–100 μM	31.5 nM	2022	[43]
MoS_2_-cucurbit[8]uril, GCE, SWV	1.25–50 μM	0.38 μM	2023	[45]
HD-CNT-fiber cross-section microelectrode, SWV	0.05–100 μM	10 nM		Present work

**TABLE 3 |:** Recovery studies for melatonin spiked in urine samples using the HD-CNT-fiber cross-section microelectrode.

Sample	Spiked Mel (μM)	Observed Mel (μM)	Recovery %	Error%

Urine	5	5.124	102.48	+2.48
	15	14.889	99.26	−0.74
	25	24.889	99.55	0.44

The RSD value for melatonin estimation was less than±3.41% for n = 3.

**TABLE 4 |:** Recovery studies for melatonin in pharmaceutical tablets using the HD-CNT-fiber cross-section microelectrode.

Sample	Actual concentration (μM)	Observed concentration (μM)	Error%

Natrol, 3 mg	5	4.94	−1.20
	10	10.24	+2.4
	15	14.44	−3.73

Walgreens, melatonin, 10 mg			
	10	10.06	+0.66
	15	14.71	−1.93
	25	25.11	+0.44

The RSD value for melatonin determination in pharmaceutical tablets was less than±3.75% for n = 3.

* Error %=[(Actual concentration - Observed concentration)/actual concentration]×100.

## Data Availability

Authors confirm that the data supporting the findings of this study are available within the manuscript and supplementary materials of this article.
