# Supplementary material for: Carbon-Nanotube Microelectrodes for Electrochemical Determination of Melatonin
Source: Electroanalysis. Author manuscript; Available in PMC 2025 Oct 22. (PMC12539611; doi:10.1002/elan.202400191)
Supplement: Suplementary Information [file NIHMS2115185-supplement-Suplementary_Information.pdf]

Electroanalysis

Supporting Information

Carbon-Nanotube Microelectrodes for Electrochemical Determination of Melatonin

Neeraj Kumar | Nilni E. Weerawarna | Noe T. Alvarez

Carbon-nanotube microelectrodes for electrochemical determination of melatonin

Neeraj Kumar, Nilni Weerawarna, *Noe T. Alvarez

***Email:** alvarene@ucmail.uc.edu

Department of Chemistry, University of Cincinnati, USA-45220

Raman spectra of vertically alinged CNT array

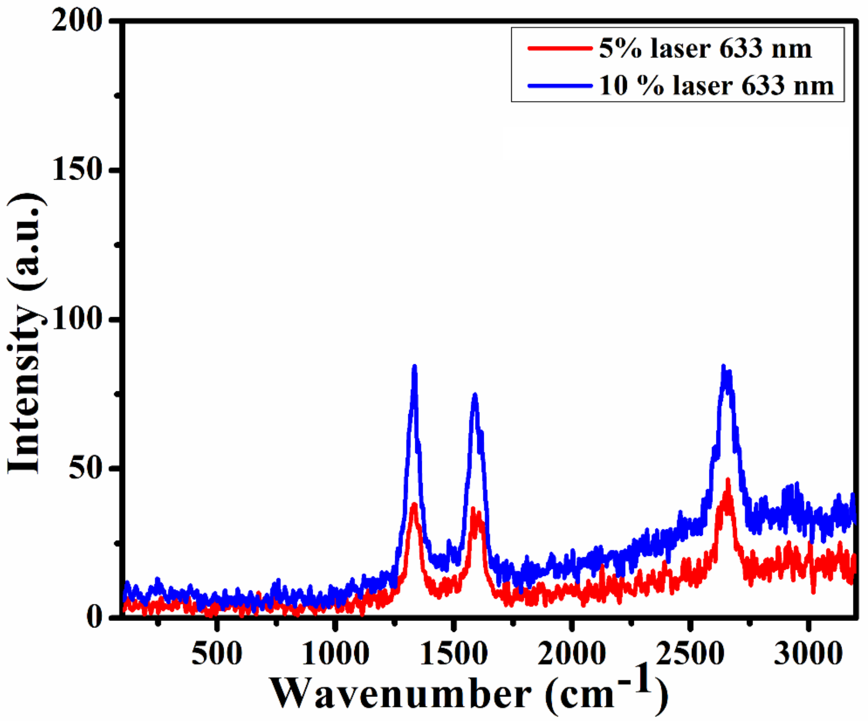

Figure S1: Raman spectra of vertically aligned CNT array recorded with 5 and 10 % power (10 sec acquisition time).

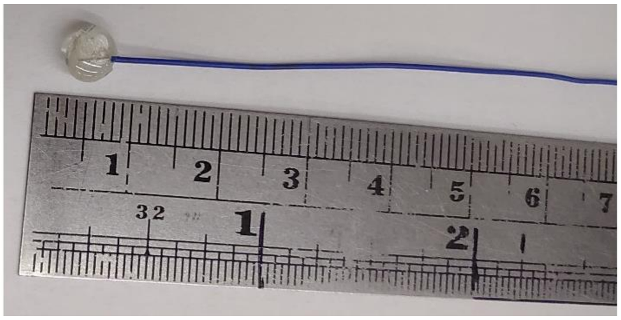

Figure S2:) A photograph of an HD-CNT-fiber cross-sections microelectrode utilized throughout this work, along with a size comparison with a lab scale.

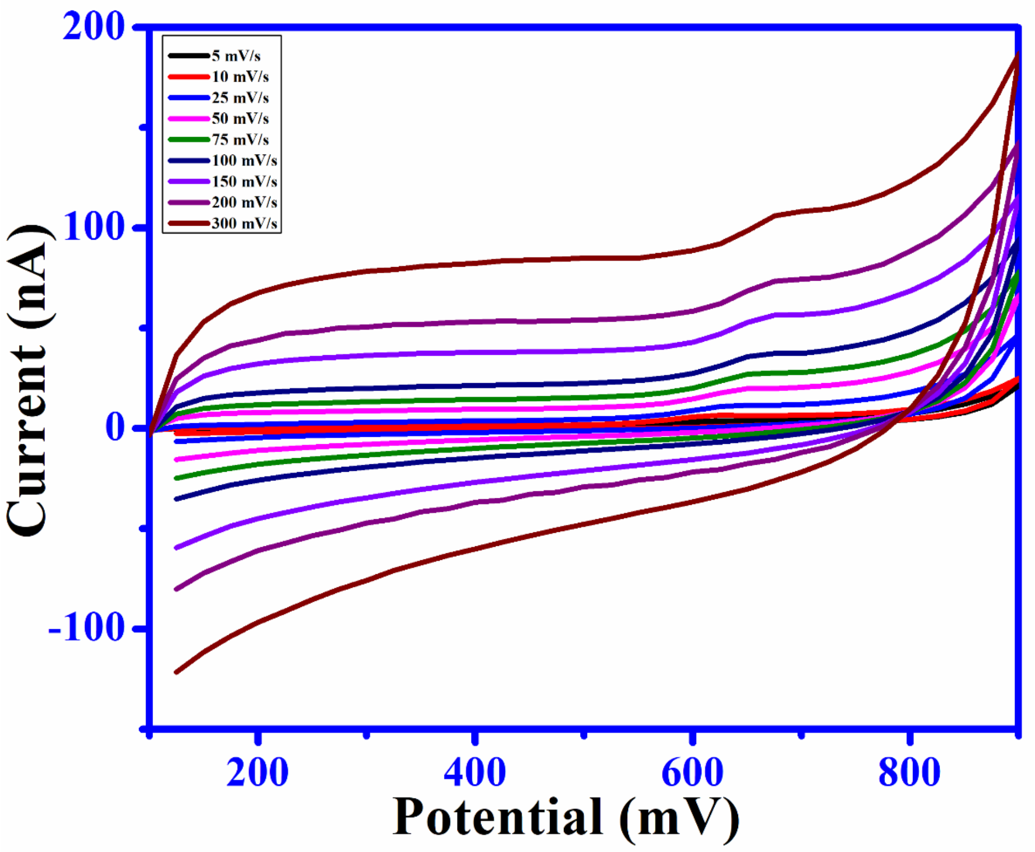

Figure S3: CV curves observed at different scan rate from 5-300 mV/s for the 10 μM Mel at cross-section microelectrode in the phosphate buffer pH 7.25.

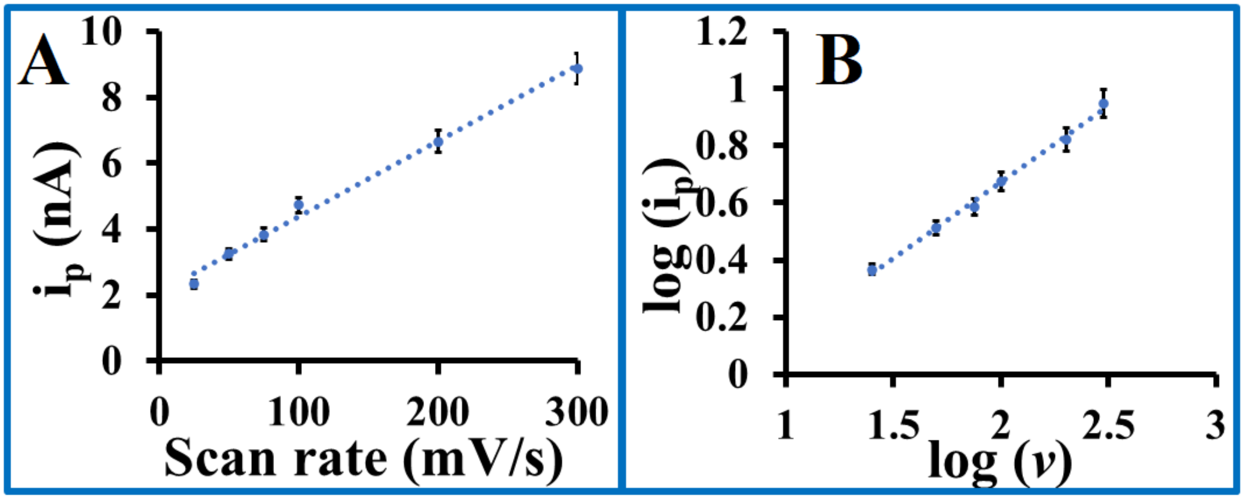

Figure S4: Variation of electro-oxidation peak current (i_p) vs. scan rate (v), and (B) log (i_p) with log (v) for 10 μM Mel using microelectrode.

Mung bean sample analysis

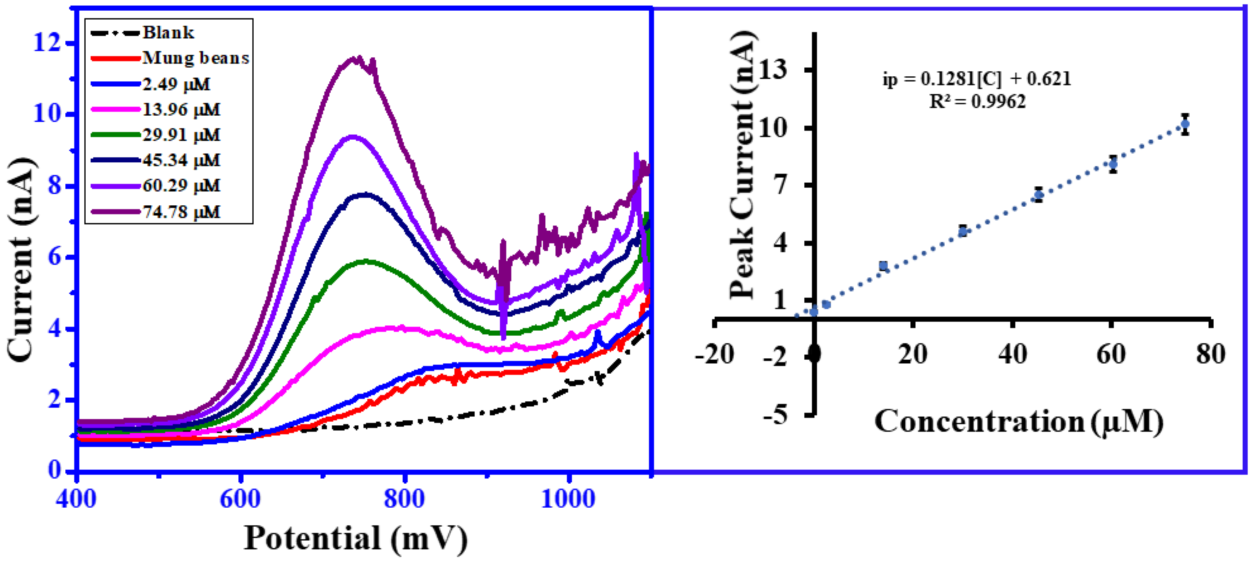

Figure S5 (A): Square wave voltammograms recorded for detection of melatonin in mung bean sample for standard addition plot, and (B) The standard addition plot for estimation of Mel in Mung Bean sample.
